# Cutaneous vascular reactivity and flow motion response to vasopressin in advanced vasodilatory shock and severe postoperative multiple organ dysfunction syndrome

**DOI:** 10.1186/cc4845

**Published:** 2006-03-07

**Authors:** Günter Luckner, Martin W Dünser, Karl-Heinz Stadlbauer, Viktoria D Mayr, Stefan Jochberger, Volker Wenzel, Hanno Ulmer, Werner Pajk, Walter R Hasibeder, Barbara Friesenecker, Hans Knotzer

**Affiliations:** 1 Department of Anesthesiology, Innsbruck Medical University, Innsbruck, Austria; 2 Department of Biostatistics and Documentation, Innsbruck Medical University, Innsbruck, Austria; 3 Department of Anesthesiology and Critical Care Medicine, Krankenhaus der Barmherzigen Schwestern, Ried im Innkreis, Austria

## Abstract

**Introduction:**

Disturbances in microcirculatory homeostasis have been hypothesized to play a key role in the pathophysiology of multiple organ dysfunction syndrome and vasopressor-associated ischemic skin lesions. The effects of a supplementary arginine vasopressin (AVP) infusion on microcirculation in vasodilatory shock and postoperative multiple organ dysfunction syndrome are unknown.

**Method:**

Included in the study were 18 patients who had undergone cardiac or major surgery and had a mean arterial blood pressure below 65 mmHg, despite infusion of more than 0.5 μg/kg per min norepinephrine. Patients were randomly assigned to receive a combined infusion of AVP/norepinephrine or norepinephrine alone. Demographic and clinical data were recorded at study entry and after 1 hour. A laser Doppler flowmeter was used to measure the cutaneous microcirculatory response at randomization and after 1 hour. Reactive hyperaemia and oscillatory changes in the Doppler signal were measured during the 3 minutes before and after a 5-minute period of forearm ischaemia.

**Results:**

Patients receiving AVP/norepinephrine had a significantly higher mean arterial pressure (*P *= 0.047) and higher milrinone requirements (*P *= 0.025) than did the patients who received norepinephrine only at baseline. Mean arterial blood pressure significantly increased (*P *< 0.001) and norepinephrine requirements significantly decreased (*P *< 0.001) in the AVP/norepinephrine group. Patients in the AVP/norepinephrine group exhibited a significantly higher oscillation frequency of the Doppler signal before ischaemia and during reperfusion at randomization. During the study period, there were no differences in either cutaneous reactive hyperaemia or the oscillatory pattern of vascular tone between groups.

**Conclusion:**

Supplementary AVP infusion in patients with advanced vasodilatory shock and severe postoperative multiple organ dysfunction syndrome did not compromise cutaneous reactive hyperaemia and flowmotion when compared with norepinephrine infusion alone.

## Introduction

Impaired microcirculatory blood flow has been identified as a key component in the pathophysiology of multiple organ dysfunction syndrome after surgery [1] and in sepsis [2]. The precise mechanisms involved remain unclear but include complex interactions between various factors: increased heterogeneity of capillary blood flow; reduced erythrocyte deformability; endothelial cell dysfunction with increased permeability and apoptosis; altered vasomotor tone; increased numbers of activated neutrophils with more neutrophil-endothelial interactions; and activation of the clotting cascade with formation of microthrombi [3]. De Backer and colleagues [2] identified impaired capillary perfusion, assessed by orthogonal polarization spectrophotometry, as an independent predictor of mortality in severe sepsis. Similarly, Sakr and colleagues [4] observed that patients who died from persistent septic multiple organ dysfunction had markedly impaired microcirculatory perfusion over time compared with surviving patients.

Although macrocirculatory parameters such as mean arterial blood pressure (MAP) and cardiac output are unreliable for predicting microcirculatory homeostasis, there is strong evidence that a certain perfusion pressure – probably a MAP above 65 mmHg if it is combined with adequate cardiac output – is a prerequisite for adequate microcirculatory blood flow [5-7]. Although vasopressor infusion alone is undoubtedly detrimental to microcirculatory blood flow, it is currently recommended that fluid resuscitation and infusion of inotropic agents to achieve adequate cardiac output be combined with vasopressor therapy in order to realize a reasonable tissue perfusion pressure [5].

However, in a small group of patients with cardiocirculatory failure, recommended standard therapy alone is not sufficient to attain a MAP necessary to maintain perfusion of an altered microcirculation.

It has been reported that supplementary infusion of arginine vasopressin (AVP) can reliably increase MAP to above 65 mmHg, even in patients with advanced cardiovascular failure who are resistant to standard treatment [8-10]. However, when AVP was continuously infused in healthy animals it resulted in severe disturbances in capillary blood flow and tissue oxygenation [11]. The effects of a supplementary AVP infusion on microcirculation in humans with severe cardiovascular failure are unknown. Exacerbation of microvascular failure by a therapeutic intervention such as vasopressor therapy would be detrimental to cell oxygenation, organ regeneration and, ultimately, patient survival. Our study group reported a 30.2% incidence of ischaemic skin lesions in patients with advanced vasodilatory shock during supplementary AVP infusion [12].

This clinical study was conducted to evaluate prospectively the cutaneous microcirculatory response to a combined infusion of AVP and norepinephrine when compared with infusion of norepinephrine alone, using laser Doppler flowmetry in patients with advanced vasodilatory shock and severe postoperative multiple organ dysfunction syndrome (Table 1).

## Materials and methods

The study protocol was approved by the institutional review board and the ethical committee of the Innsbruck Medical University, Innsbruck, Austria. Written informed consent was obtained from each patient's next of before randomization.

We prospectively enrolled 18 critically ill patients suffering from severe multiple organ dysfunction syndrome following cardiac or major surgery, with a MAP below 65 mmHg despite adequate volume resuscitation, and norepinephrine requirements in excess of 0.5 μg/kg per minute. Patients with peripheral arterial vascular occlusive disease or insulin-dependent diabetes mellitus were excluded. All patients were subjected to invasive monitoring including central venous, arterial and pulmonary artery catheter. Fluid resuscitation was performed using colloid solutions until the stroke volume index could not be increased further by volume loading. The corresponding pulmonary capillary wedge pressure was used as a therapeutic target for further fluid resuscitation. If stroke volume index remained below 25 ml/min per m^2^, cardiac index remained below 2 l/min per m^2^, or mixed venous saturation remained below 65%, then a continuous infusion of milrinone was started at doses ranging from 0.3 to 0.7 μg/kg per minute. All patients were mechanically ventilated and received analgesic and sedative drugs (for instance, continuous infusion of sufentanil and midazolam). There was no difference in the dosage of analgesic and sedative drugs between groups. No patient was paralyzed at the time of study measurements.

Upon inclusion in the study, patients were randomly assigned to an AVP/norepinephrine or a norepinephrine-only group, guided by a random number generating computer program. The study was not blinded. In patients in the AVP/norepinephrine group, supplementary AVP (Pitressin^®^; Pfizer, Karlsruhe, Germany) was infused at a continuous rate of 4 IU/hour; no bolus injections were administered. Norepinephrine infusion was adjusted to maintain MAP above 65 mmHg. In patients in the norepinephrine group, a MAP above 65 mmHg was achieved by adjusting the norepinephrine dosage.

Age, sex, past medical history (arterial hypertension, congestive heart failure, coronary heart disease, chronic pulmonary disease, chronic renal disease, non-insulin-dependent diabetes mellitus) and intensive care unit mortality were recorded in all patients. A modified Goris multiple organ dysfunction syndrome score [13] was calculated from the worst clinical data recorded before study entry; MAP, cardiac index, systemic oxygen transport and consumption variables, norepinephrine and milrinone requirements, as well as pH and arterial lactate concentrations were documented immediately before and 1 hour after randomization.

Cutaneous microcirulatory measurements were performed using a laser Doppler flowmeter (Periflux 4001; Perimed, Järfälla, Sweden). A fiberoptic guidewire (PF407; Perimed) conducts laser light with a wavelength of 770–790 nm to tissue (catchment volume about 1 mm^3^) and carries back-scattered light to a photodetector. Calibration of the photodetector was performed using the manufacturer's calibration kit. The surface of a white compact synthetic material was used to set the zero value for arbitrary perfusion units, whereas the second value of the calibration curve (perfusion units = 250) was derived by measurement in a motility standard fluid (Perimed). The electrode was placed on the volar aspect of the forearm and held in place by a thin transparent silicon rubber patch. This patch remained in the same place during the study period in order to reduce short-term intra-individual variation in laser Doppler flowmetry, which was reported to be 25.4%. Inter-individual variation was observed to be 36% [14]. Using this setting, laser Doppler flowmetry has been shown to be an suitable technique for evaluating both reactive hyperaemia [15] and oscillatory changes in vascular tone [16].

**Figure 1:**
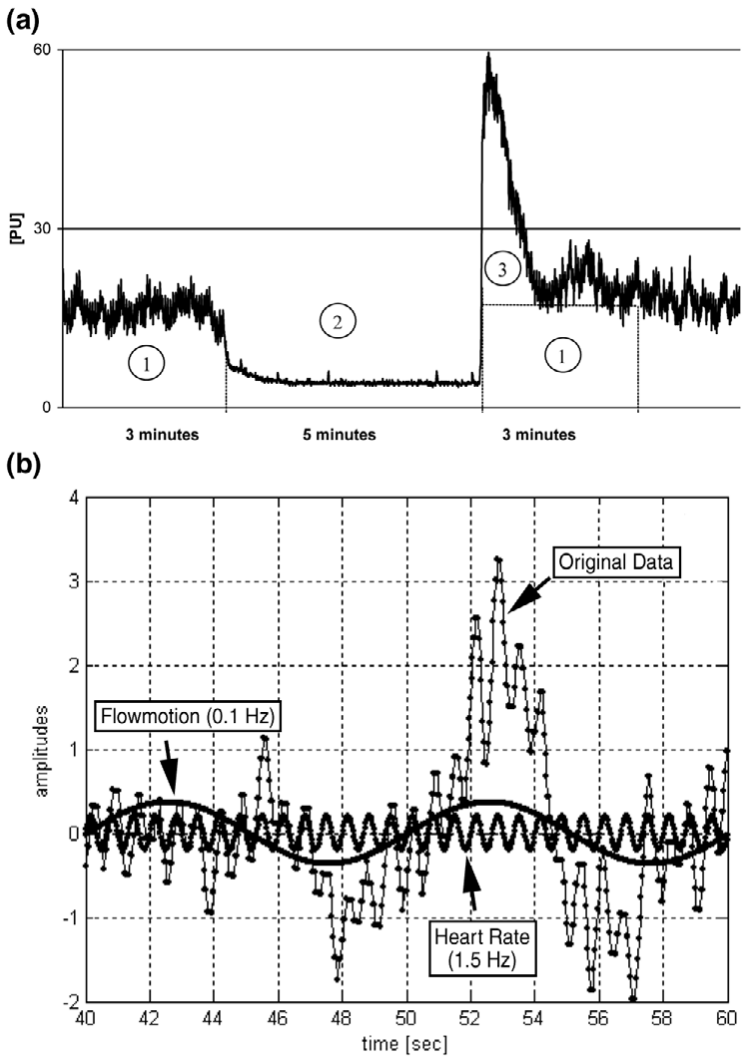
Principles of measurement of reactive hyperaemia and flowmotion using laser Doppler flowmetry. **(a) **Original Doppler tracing in a patient over a time period of 11 minutes (3 minutes pre-ischaemic, 5 minutes ischaemia, 3 minutes reperfusion time). The area under the curve (AUC) was calculated for each time interval. 1 = AUC pre-ischemic; 2 = AUC during ischaemia; 3 = AUC during reactive hyperaemia; 1 + 3 = AUC during reperfusion. **(b) **Twenty seconds of an original Doppler tracing with superimposed fast Fourier transformation. Computed analysis of the original plot reveals two oscillations with different frequencies representing heart rate and flowmotion.

Baseline measurements included the area under the curve (AUC) of the Doppler signal (given in perfusion units) and oscillatory changes (oscillations/min) of the Doppler signal over 3 minutes (pre-ischaemic). Afterward, forearm ischaemia was produced by wrapping a sphygomanometer cuff around the arm over the brachial artery and inflating it to 300 mmHg for 5 minutes. During the first 3 minutes of reperfusion, the AUC of the Doppler signal as well as oscillatory changes in the Doppler signal were measured (reperfusion). The relative magnitude of reactive hyperaemia, as a percentage, was determined using the formula 100 × (AUC_reperfusion _- AUC_pre-ischaemic_)/AUC_pre-ischaemic _(Figure 1), in order to compensate for individual differences in forearm skin vascularization. These sets of measurements were performed in all patients immediately before and 1 hour after randomization.

For flowmotion analysis, the Doppler signal tracing was divided into nine blocks of 20 seconds each before induction of ischaemia and during reperfusion measurements (Figure 1). Fast Fourier transformation analysis was performed for single blocks to obtain a quantitative description of the main oscillatory frequency component. After computing a power spectrum for each block, the medians were averaged to give the final power spectrum for pre-ischaemic and reperfusion oscillations. Frequencies corresponding to heart rate or mechanical ventilation were discarded. Mean values of oscillation were used for statistical comparisons.

### Statistical analysis

The primary objective of the study was to evaluate differences in the AUC of the Doppler signal and the reactive hyperaemic response to forearm ischaemia between AVP/norepinephrine and norepinephrine groups. The secondary study objective was to evaluate differences in the oscillation frequency of the Doppler signal between groups.

Demographic and clinical data were compared using Student's *t* test or χ^2 ^tests, as appropriate (SPSS^® ^11.0 for Windows; SPSS Inc., Chicago, IL, USA). To evaluate differences in haemodynamic, acid-base and microcirculatory variables between groups at baseline and over time, an unpaired Student's *t* test was performed. For variables that did not fulfil the normality assumption (AUC pre-ischaemic, magnitude of reactive hyperaemia, oscillation of the Doppler signal before ischaemia and during reperfusion), nonparametric tests (Mann-Whitney *U *rank sum tests) were applied. For detection of changes in single variables between the two measurements in each of the study groups, a paired Student's *t* test was used. The main effects between groups and within repeated measurements were considered to indicate statistical significance if the *P *value was below 0.05. All data are expressed as mean values ± standard deviation, unless indicated otherwise.

## Results

Cardiovascular function could not be stabilized adequately by incremental dosages of norepinephrine in one patient randomly assigned to the norepinephrine group, but MAP could be restored with supplementary AVP infusion. This patient was therefore switched to the AVP/norepinephrine group for statistical evaluation.

Of all study patients, 55% (10 out of 18) were admitted to the intensive care unit after heart surgery. The other eight patients were admitted because of severe systemic inflammatory response syndrome or sepsis after major abdominal surgery (*n *= 6) or noncardiac surgical thoracic surgery (*n *= 2). The time between admission to the intensive care unit and study entry was between 24 and 36 hours in all patients.

Between AVP/norepinephrine (*n *= 10) and norepinephrine patients (*n *= 8), there were no differences in age (70.5 ± 8.5 years versus 67 ± 7.1 years; *P *= 0.196), male sex (60% versus 62.5%; *P *= 1), pre-existing morbidity (*P *= 0.784), multiple organ dysfunction syndrome score (12.3 ± 1.1 versus 12.2 ± 0.4; *P *= 0.84), and intensive care unit mortality (80 versus 87.5%; *P *= 1).

Table 1 presents macrocirculatory, acid-base, and laser Doppler flowmetry derived variables in the AVP/norepinephrine and norepinephrine groups. Patients receiving AVP/norepinephrine had significantly higher MAP and milrinone requirements than did those in the norepinephrine group. During the observation period, MAP increased significantly (*P *< 0.001) and norepinephrine requirements significantly decreased (*P *< 0.001) in the AVP/norepinephrine group. There were no further differences in haemodynamic or acid-base variables between groups or over time.

No differences in the pre-ischaemic AUC of the Doppler signal and in the magnitude of reactive hyperaemia occurred between groups before and 1 hour after randomization. Patients receiving AVP/norepinephrine had a higher oscillation frequency of the Doppler signal before ischaemia and during reperfusion at study inclusion than did patients receiving norepinephrine alone. One hour after study drug infusion there was no difference in the oscillation frequency before ischaemia and during reperfusion between groups when adjusted for baseline differences.

## Discussion

As described previously [10,11], supplementary AVP infusion was beneficial in that it improved MAP and allowed norepinephrine dosage to be reduced compared with norepinephrine infusion alone. AVP therapy neither reduced the pre-ischaemic AUC of the Doppler signal nor reduced the magnitude of the reactive hyperaemic response at up to five minutes of forearm ischaemia. Additionally, there was no difference in the response of the oscillation frequency detected by laser Doppler flowmetry.

Reactive hyperaemia is the transient increase in organ blood flow that occurs after ischaemia. In general, the ability of an organ to exhibit reactive hyperemia reflects the autoregulative capacity of its microcirculation [1]. Representing the proportion of recruitable capillaries, arterioles, and small arteries, reactive hyperaemia was found to be significantly attenuated in patients with shock [1,17,18]. In this study, the combined infusion of AVP and norepinephrine did not change the pre-ischaemic AUC of the Doppler signal and the magnitude of reactive hyperaemia when compared with patients receiving norepinephrine infusion alone. Thus, even though AVP significantly increased MAP, its strong vasoconstrictive effects clearly did not further compromise autoregulation of the cutaneous microcirculation in severe postoperative multiple organ dysfunction syndrome. Nonetheless, it cannot be excluded that a higher MAP, as seen in the AVP/norepinephrine group after 1 hour, could have concealed a potentially deleterious effect of AVP on the reactive hyperaemic response. An unchanged AUC of the laser Doppler signal before induction of forearm ischaemia indicates that AVP did not significantly reduce blood flow in the skin tissue volume examined.

Vasomotion is the oscillation of vascular tone that is generated from within the vascular wall; it is not a consequence of heart beats, respiration, or neuronal input [16]. Initiation of vasomotion can be observed during hypoxia, tissue hypoperfusion, acidosis, and during AVP infusion in the skin of healthy hamsters [11]. Because blood vessels with an oscillating diameter have higher conductance than do vessels with a constant diameter of the same average width [19], it was suggested that vasomotion might reflect a rescue mechanism in conditions of tissue hypoxia in order to ensure adequate tissue oxygenation [16]. In this study population with advanced cardiovascular failure and severe postoperative multiple organ dysfunction syndrome, combined infusion of AVP and norepinephrine did not increase oscillation frequency of the Doppler signal when compared with norepinephrine infusion alone. At least in these patients, this finding may indicate that AVP/norepinephrine does not result in further deterioration in microcirculatory oxygen supply in skin.

These findings are in striking contrast to the results of a physiologic animal experiment, in whihc the effects of AVP on the microcirculation were analyzed in the skinfold of healthy hamsters [11]. In that model, doses of AVP similar to those used in clinical practice (4 IU/hour) resulted not only in significant reductions in skin blood flow and oxygenation but also in an increase in vasomotion frequency. Aside from species dependent variations, differences between physiological and pathophysiological states appear to be important determinants of the effects of AVP on cutaneous microcirculatory flow. Although AVP induces substantial vasoconstriction in normally contracted arterioles in healthy animals, the effects of AVP in an excessively vasodilated microcirculation [20] with decreased susceptibility to AVP [21-23] appear to be significantly different.

Thus far only two case reports have been reported that describe the microcirculatory response to AVP in patients with advanced shock states. Similar to our data, no further deterioration in microcirculatory flow in the sublingual tissue, as assessed by orthogonal polarization spectrophotometry, was detected during supplementary AVP infusion by Dubois and colleagues [24]. Interestingly, in this patient administration of AVP actually appeared to improve microcirculatory flow because of an increase in the proportion of perfused capillaries [24]. Recently, Boerma and colleagues [25] used the same technique in a patient with terminal septic shock receiving a bolus injection of terlipressin. In contrast to the first case report and our findings, a dramatic decrease in small vessel numbers and, ultimately, a complete standstill in sublingual capillary flow occurred.

In their patient, Boerma and colleagues [25] further observed clinically evident hypoperfusion of the distal extremities, a decreased peripheral perfusion index, as well as a substantial increase in the central-to-toe temperature difference (from 1.7 to 13.4°C) after terlipressin injection. Similarly, our study group reported a 30.2% incidence of ischaemic skin lesions in patients with advanced vasodilatory shock during supplementary AVP infusion [12]. However, in a prospective, controlled study, ischaemic skin lesions occurred at a comparable rate in severe shock patients with or without AVP infusion, indicating that ischaemic lesions of the skin rather reflect severity of the underlying microcirculatory failure than direct adverse side effects of AVP infusion [8]. Taking these limited data together, it seems that several factors (for example, dose, the vasopressin analogue used, circulating blood volume and cardiac output, severity of illness and microcirculatory dysfunction) as well as genetic factors determine the microcirculatory response to AVP-induced vasoconstriction in advanced cardiovascular failure.

When interpreting the results of this study, important limitations must be considered. First, because measurements taken in the present study can only be interpreted as a response of the skin vessels to AVP, these results may not be extrapolated to other vascular beds. However, in contrast to most 'internal' organs, vasoconstriction in skin is mediated almost exclusively by receptors [26]; therefore, one could assume that the vasoconstrictor response to any vasopressor agent would be most pronounced in skin. Second, using laser Doppler flowmetry, heterogeneity in microcirculatory blood flow, a consistent finding in both endotoxaemic animal models [27,28] and human studies [2,4], cannot be assessed. Therefore, unchanged microcirculatory parameters during supplementary AVP infusion, as indicated by laser Doppler flowmetry in this study population, does not exclude altered heterogeneity in microcirculatory flow. Third, although no data exist on the effects of milrinone and vasomotion, it cannot be ruled out that the significantly higher milrinone dosages in the AVP/norepinephrine groups mitigated a decrease in reactive hyperaemia and amplification of vasomotion. Finally, it is conceivable that if measurements had been repeated 12 or 24 hours after randomization, the microcirculatory response might have been different from that reflected by measurements taken 1 hour after randomization. Nonetheless, relevant changes in reactive hyperaemia and arteriolar vasomotion are known to occur within very short periods of time [29].

## Conclusion

Supplementary AVP infusion in patients with advanced vasodilatory shock and severe postoperative multiple organ dysfunction syndrome did not compromise cutaneous reactive hyperaemia and flowmotion when compared with norepinephrine infusion alone.

## Key messages

• 	Supplementary AVP infusion in patients with advanced vasodilatory shock and severe postoperative multiple organ dysfunction syndrome did not decrease cutaneous reactive hyperaemia and flowmotion when compared with norepinephrine infusion alone.

## Abbreviations

AUC = area under the curve; AVP = arginine vasopressin; MAP = mean arterial pressure.

## Competing interests

VW has received a grant from Aguettant Laboratories, Lyon, France, a company that has applied for registration of vasopressin with the European authorities. There is no personal conflict of interest.

## Authors' contributions

GL conceived the study protocol, participated in its design and coordination, carried out bedside measurements and documentation, and drafted the manuscript. MWD conceived the study protocol, participated in its design and coordination, carried out bedside measurements and documentation, performed the statistical analysis, and helped to draft the manuscript. KHS participated in the design of the study and performed the calculations for the microcirculatory measurements. VDM conceived the study, helped to carry out bedside measurements and documentation, and contributed to the drafting of the manuscript. SJ conceived the study, and helped to carry out bedside measurements and documentation. VW participated in the study design and its coordination, and helped to draft the manuscript. HU performed the statistical analysis and helped to interpret the data. WP conceived the study, and helped to carry out bedside measurements and documentation. WRH conceived the study protocol, and helped to interpret the data and to draft the manuscript. BF helped to interpret the data and to draft the manuscript. HK conceived the study protocol, participated in its design and coordination, carried out bedside measurements and documentation, and helped to draft the manuscript. All authors read and approved the final version of the manuscript.

## Figures and Tables

**Table 1:** Changes in haemodynamic variables, acid-base status, reactive hyperaemia, and vasomotion

Parameters	Group	Baseline	1 hour	*P*
MAP (mmHg)	AVP/NE*	64 ± 5	81 ± 12	0.047^†^
	NE	64 ± 9	66 ± 13	
CI (l/min per m^2^)	AVP/NE	4.1 ± 1.2	3.8 ± 1.1	0.152
	NE	3.5 ± 0.9	3.5 ± 0.8	
DO_2_I (ml/min/m^2^)	AVP/NE	571 ± 140	526 ± 130	0.151
	NE	482 ± 111	493 ± 119	
VO_2_I (mL/min/m^2^)	AVP/NE	155 ± 39	157 ± 35	0.077
	NE	131 ± 35	137 ± 41	
SvO_2 _(%)	AVP/NE	72 ± 6	66 ± 13	0.583
	NE	71 ± 9	70 ± 10	
NE requirements (μg/kg per min)	AVP/NE*	0.75 ± 0.31	0.51 ± 0.19	0.36
	NE	0.65 ± 0.23	0.76 ± 0.18	
Milrinone requirements (μg/kg per min)	AVP/NE	0.44 ± 0.15	0.44 ± 0.16	0.025^†^
	NE	0.31 ± 0.2	0.29 ± 0.19	
pH	AVP/NE	7.30 ± 0.12	7.30 ± 0.12	0.355
	NE	7.33 ± 0.1	7.32 ± 0.08	
Lactate (mmol/l)	AVP/NE	5.9 ± 6.7	6.6 ± 6.3	0.548
	NE	7.6 ± 6.1	7.5 ± 5.6	
AUC pre-ischaemic	AVP/NE	21.2 ± 9.4	16.7 ± 7.7	0.089
	NE	15.3 ± 10.2	15.3 ± 9.0	
Magnitude of RH (%)	AVP/NE	64 ± 57.2	70 ± 47.5	0.56
	NE	49.4 ± 24.8	48.7 ± 41.7	
ODS pre-ischaemic (oscillations/min)	AVP/NE^‡^	15.73 ± 5.15	14.38 ± 7.31	0.055^§^
	NE	7.49 ± 3.2	10.7 ± 3.81	
ODS reperfusion (oscillations/min)	AVP/NE^‡^	14.38 ± 4.85	13.78 ± 9.17	0.601^§^
	NE	8.99 ± 3.92	10.7 ± 5.7	

Data are expressed as mean values ± standard deviation. AUC, area under the curve; AVP, arginine vasopressin; CI, cardiac index; DO_2_I, systemic oxygen transport index; MAP, mean arterial pressure; NE, norepinephrine; ODS, oscillation of the Doppler signal; RH, reactive hyperaemia; SvO_2_, mixed venous oxygen saturation; VO_2_I, systemic oxygen consumption index. *significant difference over time; ^†^, significant difference between groups; ^‡^, significant difference at baseline between groups; ^§^, corrected for baseline differences.
